# Supplementary material for: Digital citizen science for ethical monitoring of youth physical activity frequency: Comparing mobile ecological prospective assessments and retrospective recall
Source: PLOS Digit Health. 2025 May 2;4(5):e0000840. doi: 10.1371/journal.pdig.0000840 (PMC12047779; doi:10.1371/journal.pdig.0000840)
Supplement: Attachment — Submitted filename: PLOS digital health review.pdf [file pdig.0000840.s001.pdf]

<u>The title:</u> I recommend reducing redundancy (e.g., "ethical surveillance" can be better phrased as "ethical monitoring") and clearly indicating the comparison between two assessment methods. Additionally, emphasizing "youth physical activity" ensures the target population is immediately identifiable.

I recommend the title: "Digital Citizen Science for Ethical Monitoring of Youth Physical Activity: Comparing Mobile Ecological Prospective Assessments and Retrospective Recall." Consider capitalizing the start letter for every word in the title.

<u>Abstract:</u>

- **Introduction (Lines 52–54):**
    - The introduction briefly mentions "digital citizen science" without providing enough context for its relevance or novelty. Readers unfamiliar with the concept might require more explanation.
    - **Recommendation:** Include a brief statement on why digital citizen science is valuable for studying youth physical activity patterns.

- **Objective (Lines 55–60):**
    - The abstract lists the three types of questionnaires used but does not explain why these specific tools were chosen or how they differ. This could leave the reader wondering about the rationale behind their selection.
    - **Recommendation:** Briefly justify the choice of the International Physical Activity Questionnaire (IPAQ), Simple Physical Activity Questionnaire (SPAQ), and Global Physical Activity Questionnaire (GPAQ).

- **Methods (Lines 61–65):**
    - The abstract mentions that youth citizen scientists were recruited, but it does not clarify the recruitment criteria or the ethical considerations for involving minors.
    - **Recommendation:** Mention the inclusion criteria and ethical protocols, such as obtaining informed consent from participants or guardians.

- **Results (Lines 66–70):**
    - The abstract states that there was a "significant difference" without providing effect sizes or confidence intervals, which are crucial for understanding the magnitude of the findings.
    - The associations identified (e.g., support from family and friends, drug-free friends) are listed without elaboration on why these factors might influence MVPA frequency differently in prospective versus retrospective assessments.
    - **Recommendation:** Include effect sizes or confidence intervals to give a clearer picture of the results. Briefly explain why certain factors were associated with prospective or retrospective MVPA frequencies.

- **Conclusion (Lines 71–74):**

- The conclusion suggests that mEPAs are a promising approach but does not mention any limitations or challenges (e.g., reliance on smartphone access, potential biases in self-reporting).
- **Recommendation:** Briefly acknowledge the limitations of using digital devices for PA surveillance among youth and suggest areas for future research.

Grammar: Some examples of suggested corrections: Other refinements are needed.

Lines 52–53:

- Physical inactivity is the fourth leading risk factor of mortality globally, which could be corrected as follows: Physical inactivity is the fourth leading risk factor for mortality worldwide.

Lines 55–56:

- As digital citizen science approaches utilizing citizen-owned smartphones to ethically obtain MVPA big data can transform physical activity (PA) surveillance..., can be corrected to Digital citizen science approaches using citizen-owned smartphones to collect MVPA data ethically have the potential to transform physical activity (PA) surveillance.

Lines 57–58:

- ...using smartphone-deployed retrospective validated surveys (adapted from the International Physical Activity Questionnaire, the Simple Physical Activity Questionnaire, and the Global Physical Activity Questionnaire)... cam be corrected to...using smartphone-deployed, validated retrospective surveys adapted from the International Physical Activity Questionnaire (IPAQ), Simple Physical Activity Questionnaire (SPAQ), and Global Physical Activity Questionnaire (GPAQ)...

Line 62:

- *A total of 68 participants (aged 13 to 21) provided complete data... could be correct to,* Sixty-*eight participants (aged 13–21) provided complete data...* (I prefer spelling out numbers at the beginning of a sentence.)

<u>Introduction:</u>

- The rationale for comparing retrospective recall with mobile ecological prospective assessments (mEPAs) is only briefly mentioned. The introduction would benefit from a clearer justification for why this comparison is crucial, especially in a youth cohort. I recommend Strengthening the rationale by providing a more detailed explanation of the limitations of retrospective recall, particularly in youth populations, and the potential advantages of using MEPs.

- The transition from conventional ecological momentary assessments to the adapted mEPAs is somewhat abrupt. The decision to modify the reporting interval is explained, but there is limited discussion on the potential trade-offs of this change (e.g., reduced real-time accuracy). You can **p**rovide a more balanced discussion of the benefits and

limitations of adapting momentary assessments to end-of-day reporting, including potential impacts on data quality.

- The use of Social Cognitive Theory is mentioned briefly without much detail. The connection between the theory and the research design could be elaborated to demonstrate how it informs the choice of assessment methods (retrospective vs. mEPAs). You must expand on the application of Social Cognitive Theory and its relevance to the selection of assessment tools and measurement of PA among youth.

- The challenges related to digital literacy differences, device access, and compliance with digital assessments are not adequately addressed. I think it's good to discuss potential barriers to implementing digital citizen science approaches, such as disparities in access to smartphones or varying levels of digital literacy among participants.

- The hypothesis is not fully integrated with the theoretical framework or the limitations of previous research. Additionally, the study aim could be more concisely stated. You can link the hypothesis to the identified gaps in the literature and provide a concise and focused statement of the study's objectives.

Line 103:

- Evidence is clear that physical activity (PA) is crucial in the prevention and management of non-communicable diseases (NCD) among youth. Can be changed to The evidence clearly indicates that physical activity (PA) is crucial for the prevention and management of non-communicable diseases (NCDs) among youth.

Line 106:

- ...evidence indicating the presence of a dose-response relationship between PA and health – the more PA that youth engage in, the greater their health benefits. This can be corrected to ...evidence suggests a dose-response relationship between PA and health; the more youth engage in PA, the greater the health benefits.

Line 110:

- Additionally, engagement in consistent PA has been found to improve sleep quality, reduce symptoms of depression and anxiety, and minimize substance use. Try this version, Engaging in regular PA has been shown to improve sleep quality, reduce symptoms of depression and anxiety, and decrease substance use.

Line 121:

- Measuring MVPA frequency enables direct comparisons with established guidelines, making it easier to assess adherence to recommended levels of PA. can be corrected to**:** Measuring the frequency of MVPA allows for direct comparisons with established guidelines, facilitating the assessment of adherence to recommended PA levels.

Line 125:

- Recall errors introduce inaccuracies in reported MVPA data, while the influence of social desirability, particularly apparent in subjective recall measures, can further distort the reliability of MVPA data. To. **:** Recall errors introduce inaccuracies in reported MVPA data, and the influence of social desirability, especially in subjective recall measures, can further compromise data reliability.

Line 133:

- Repeated prospective measures can address these deficiencies of existing methodologies, particularly when deployed via citizen-owned digital devices to minimize recall bias and enhance collection of environmental factors that influence MVPA. Can be **:** Repeated prospective measures can address the limitations of existing methodologies, especially when implemented through citizen-owned digital devices, reducing recall bias and improving the capture of environmental factors affecting MVPA.

Line 171:

- To address these concerns, we modified the momentary assessments to encompass a longer reporting interval (i.e., end of day), following Shiffman et al. can be corrected: To address these issues, we adapted the momentary assessments to include a longer reporting interval (i.e., end of day), following the approach of Shiffman et al.

Methodology:

1. **Study Design:**
   - The explanation of the Smart Platform's dual interface (citizen and scientist) could be clearer. Additionally, the ethical considerations of using citizen-owned smartphones, particularly for youth, are not thoroughly discussed.
   - I recommend to Provide a brief discussion on the potential ethical issues related to digital surveillance and consent, especially for minors. Clarify how data privacy is maintained in the Smart Platform.
2. **Participants and Recruitment:**
   - The use of convenience sampling limits the generalizability of the findings. Additionally, implied informed consent from parents of younger participants (aged 13-16) may raise ethical concerns.
   - I recommend to **:** Acknowledge the limitations of using convenience sampling. Provide more details on how implied consent was obtained and any steps taken to ensure ethical compliance.
3. **Measures and Data Collection:**
   - The potential biases in self-reported data, such as recall bias and social desirability bias, are not addressed adequately. The decision to use end-of-day reporting for mobile ecological prospective assessments (mEPAs) should be discussed more critically.
   - I recommend mentioning the potential biases in self-reported measures and justify using end-of-day reporting for mEPAs as a strategy to mitigate compliance issues.
4. **Ethical Considerations:**

- The use of incentives could introduce selection bias. There is limited discussion on how the ethical implications of real-time digital monitoring were addressed, particularly for vulnerable youth populations.
- I recommend to **:** Include a brief note on how incentives were balanced with ethical considerations to avoid coercion. Discuss any measures taken to ensure participants were aware of their rights and data usage.

5. **Measurement of Independent Variables:**
   - The dichotomization of responses (e.g., peer support, strength training) may lead to a loss of nuanced information, potentially reducing the variability and depth of analysis.
   - **Recommendation:** Consider explaining the rationale for dichotomizing these variables. A sensitivity analysis could be conducted to evaluate the impact of this decision on the study outcomes.
6. **Data Collection and Risk Management:**
   - Although risk management is addressed, there is limited discussion on how data security was maintained in cases where participants chose to upload data over Wi-Fi or when their phones were plugged in. The use of a hash algorithm for encryption is mentioned, but further details on the specific method (e.g., SHA-256) would strengthen the description.
   - **Recommendation:** Provide additional details on the encryption method used and discuss potential risks associated with data transfer, especially over public Wi-Fi.
7. **Inclusion Criteria and Data Analyses:**
   - It does not address how missing data were handled or the implications of excluding cases with missing data. Additionally, the decision to use Poisson regression for count data should be justified further, considering potential overdispersion.
   - **Recommendation:** Clarify the method used for handling missing data (e.g., listwise deletion, imputation) and provide a rationale for the choice of Poisson regression, including any steps taken to check for overdispersion.
8. **Covariate Selection:**
   - The relationship between these covariates and the outcome (MVPA frequency) is not fully explained. The potential confounding effects of unmeasured variables (e.g., socioeconomic status) are not discussed.
   - **Recommendation:** Provide a brief explanation of why these specific covariates were chosen and discuss any limitations related to unmeasured confounders.

Grammatical Recommendations:

1. **Line 191:**
   - ...in Regina, an urban centre located in the Canadian prairie province of Saskatchewan. I recommend to this change ...in Regina, an urban center in the Canadian prairie province of Saskatchewan.
2. **Line 199:**
   - Ethics approval for the Smart Platform was granted by the Research Ethics Board of the University of Regina and Saskatchewan. I recommend to this change Ethical approval for the Smart Platform was granted by the Research Ethics Boards of the University of Regina and Saskatchewan .
3. **Line 212:**

- Through the app, participants reported their MVPA data, behavioural, contextual, demographic and social factors influencing their PA behaviour. I recommend to this change Through the app, participants reported their MVPA data, as well as behavioral, contextual, demographic, and social factors influencing their PA behavior.

4. **Line 218:**
   - Additionally, they were asked to provide responses to mEPAs from day 1 to day 8, including weekdays and weekends through an in-app time-triggered nudge. I recommend to this change They were also prompted to provide responses to mEPAs from day 1 to day 8, including both weekdays and weekends, via an in-app time-triggered nudge.
5. **Line 230:**
   - By conducting a sample size calculation at a 90% confidence level with a 5% margin of error, it was determined that a sample size of 273 participants was required for the study. I recommend to this change A sample size calculation at a 90% confidence level with a 5% margin of error determined that 273 participants were required.
6. **Line 238:**
   - During these recruitment sessions, the research team described the study, demonstrated how to use the custom-built app, which included demonstrations of completing mEPAs among other tasks. I recommend to this change During these recruitment sessions, the research team explained the study, demonstrated how to use the custom-built app, including how to complete mEPAs and other tasks.
7. **Line 244:**
   - Implied informed consent was obtained from parents and caregivers of participants who were between 13 and 16 years old prior to the in-person scheduled recruitment sessions. I recommend to this change Implied informed consent was obtained from parents and caregivers of participants aged 13 to 16 before the in-person recruitment sessions.
8. **Line 251:**
   - The retrospective MVPA data were collected through a survey adapted from three validated self-reported measures: the international physical activity questionnaire, the simple physical activity questionnaire, and the global physical activity questionnaire. I recommend to this change Retrospective MVPA data were collected using a survey adapted from three validated self-reported measures: the International Physical Activity Questionnaire (IPAQ), Simple Physical Activity Questionnaire (SPAQ), and Global Physical Activity Questionnaire (GPAQ).
9. **Line 309:**

   - Peer support for MVPA was captured with the question: “How many of your closest friends are physically active?” can be changed to**:** Peer support for MVPA was assessed with the question: “How many of your closest friends are physically active?”

- **Line 318:**

  - How many times in a week an adult in your household encourages PA. can be changed to: How many times per week does an adult in your household encourage PA?

10. **Line 324:**
    - Youth were asked "How many of your closest friend are committed to drug-free." can be changed to: Youth were asked, "How many of your closest friends are committed to being drug-free?"
11. **Line 332:**
    - Youth were asked: "To the best of your knowledge, how far is your school from home." To **:** Youth were asked, "To the best of your knowledge, how far is your school from home?"
12. **Line 338:**
    - Gender was captured by asking participants "What is your gender?", with five response options: "male", "female", "transgender", "other (please specify)", and "prefer not to disclose". to**:** Gender was assessed with the question, "What is your gender?" Response options included: "male," "female," "transgender," "other (please specify)," and "prefer not to disclose."
13. **Line 363:**
    - On how many days in the last 7 days did you do exercises to strengthen or tone your muscles? To: On how many days in the past 7 days did you engage in exercises to strengthen or tone your muscles?
14. **Line 385:**
    - Chi-square tests were used to test the significance difference in a proportion of independent variables included in this study. To **:** Chi-square tests were used to assess the significance of differences in the proportions of independent variables included in this study.
15. **Line 391:**
    - Covariates including peer and family support, strength training, distance and friends' drug-free were included in this study based in previous related literatures. To: Covariates, including peer and family support, strength training, distance, and friends' commitment to being drug-free, were included based on previous related literature.

Results:

1. **Sample Size and Data Completeness:**
    - The drastic reduction in sample size (from 808 to 68) due to incomplete data raises concerns about potential selection bias. The small final sample size may limit the generalizability of the findings and reduce statistical power.
    - Try to: Discuss the implications of the reduced sample size on the study's external validity. Consider addressing any potential biases introduced by excluding participants with incomplete data.
2. **Summary Statistics and Descriptive Analysis:**
    - The results lack a comparison of the demographic characteristics between the initial sample (N=436) and the final sample (N=68). This omission makes it difficult to assess whether the final sample is representative of the larger group.
    - I suggest including a comparison of key demographics between the initial and final samples to evaluate representativeness and potential selection bias.
3. **Outcome Variables and Statistical Tests:**
    - The authors do not provide effect sizes for the Wilcoxon signed-rank test, making it difficult to assess the practical significance of the observed differences.

- I suggest Reporting the effect size (e.g., Cohen's d or rank-biserial correlation) for the Wilcoxon signed-rank test to provide a better understanding of the magnitude of the difference.

4. **Poisson Regression Analysis:**
   - There is limited discussion on the issue of potential overdispersion in the Poisson regression models, which could affect the validity of the results. Additionally, the interpretation of some regression coefficients (e.g., negative associations with family support for daily MVPA) could be better contextualized.
   - So, I suggest addressing potential overdispersion by reporting the dispersion parameter and, if necessary, using a negative binomial regression model. Provide more context and interpretation for unexpected findings, such as the negative association between daily family support and prospective MVPA.
5. **Presentation of Results:**
   - The tables are dense and could benefit from more explicit labeling and explanations of abbreviations (e.g., mEPA, Ref.). Additionally, some results (e.g., peer support for MVPA) are counterintuitive and require further discussion.
   - **Recommendation:** Simplify the tables by using more evident labels and footnotes to explain abbreviations. Include a brief discussion to address counterintuitive findings, such as why youth with active friends reported lower MVPA.

Grammatical Corrections

1. **Line 396:**
   - This study involved the participation of 808 youth (13-21 years); however, when the cross-sectional survey was deployed at baseline, 436 participants provided complete data using the retrospective measure for capturing MVPA.
   - **Correction:** This study included 808 youth (aged 13-21 years); however, only 436 participants provided complete data using the retrospective MVPA measure at baseline.
2. **Line 401:**
   - Youth were predominantly males (40.30%) of youth, with 56.72% being females, and 2.98% of them reported being transgender, other, or preferring not to reveal.
   - **Correction:** The participants were predominantly female (56.72%), with 40.30% identifying as male, and 2.98% identifying as transgender, other, or preferring not to disclose.
3. **Line 416:**
   - Youth reported engaging in MVPA for an average of 5.06 times per week retrospectively and 2.10 times per week prospectively.
   - **Correction:** Youth reported an average MVPA frequency of 5.06 times per week using the retrospective measure and 2.10 times per week using the prospective (mEPA) measure.
4. **Line 433:**

- With respect to family support for MVPA, youth with a family member who encouraged them to participate in PA 5 to 6 times per week reported a higher frequency of MVPA than youth whose family members encouraged MVPA 4 times or less per week.
- **Correction:** Regarding family support for MVPA, youth whose family members encouraged PA 5 to 6 times per week reported a higher MVPA frequency compared to those whose family members encouraged PA 4 times or less per week.

5. **Line 448:**
    - Youth whose distance from school to their home was 5 km or more reported a higher frequency of MVPA in comparison to youth whose school is less than 5 km from their homes.
    - **Correction:** Youth who lived 5 km or more from their school reported a higher frequency of MVPA compared to those who lived less than 5 km from their school.
6. **Line 453:**
    - The Poisson regression analyses showing the association between retrospective MVPA frequency (model 1) and prospective MVPA frequency (model 2) with sociodemographic and contextual factors are presented in Table 3.
    - **Correction:** Table 3 presents the Poisson regression analyses showing the associations between sociodemographic and contextual factors with retrospective MVPA frequency (model 1) and prospective MVPA frequency (model 2).

Discussion Section

1. **Main Findings and Interpretation:**
    - The section could benefit from a more detailed exploration of the implications of these differences for public health practice. Additionally, the potential limitations of both retrospective and prospective methods are not thoroughly discussed.
    - **Recommendation:** Include a brief discussion on the practical implications of using mEPAs for MVPA monitoring in youth, especially in terms of informing public health guidelines. Address the limitations of both assessment methods, including potential biases and challenges related to participant compliance.
2. **Comparison with Previous Studies:**
    - Some comparisons, particularly those related to social support factors (e.g., peer influence), are only briefly mentioned without adequate explanation. The authors do not sufficiently explore why some of their findings (e.g., negative association with peer support) contradict previous studies.
    - **Recommendation:** Provide a more detailed explanation for the unexpected findings, such as the negative association between having active friends and MVPA frequency. Consider discussing potential underlying mechanisms, such as social comparison effects or differences in the sample population.
3. **Theoretical and Practical Implications:**
    - While the theoretical implications are addressed, the practical implications for interventions or public health programs are not sufficiently explored. The discussion could benefit from specific recommendations on how the findings could be applied in real-world settings.

- **Recommendation:** Include concrete suggestions for policymakers, educators, and public health practitioners on how to utilize mEPAs for monitoring and promoting physical activity among youth.

4. **Limitations of the Study:**
   - The section does not fully address the limitations related to the small final sample size (N=68) and its impact on the generalizability of the findings. Additionally, the potential challenges of using smartphone-based assessments, such as digital literacy disparities, are not discussed.
   - **Recommendation:** Clearly state the limitations related to the reduced sample size and potential selection bias. Discuss the challenges of using digital tools for data collection, especially among diverse youth populations with varying levels of access and digital skills.
5. **Future Research Directions:**
   - The suggestions for future research are somewhat general and could be made more specific. For example, the discussion does not specify which types of interventions or study designs might be effective in addressing the gaps identified.
   - **Recommendation:** Propose specific research questions or study designs that could address the limitations of the current study, such as randomized controlled trials to test the effectiveness of mEPAs in promoting physical activity.

Grammatical Corrections

1. **Line 488:**
   - This study was carried out to ascertain if there is a significant difference between the frequency of MVPA assessed through a digitally deployed retrospective survey...
   - **Correction:** This study aimed to determine whether there is a significant difference in the frequency of MVPA assessed using a digitally deployed retrospective survey...
2. **Line 496:**
   - The main finding of the study was that there was a significant difference between the frequency of MVPA reported retrospectively and the frequency of MVPA reported via mEPAs.
   - **Correction:** The main finding of the study was a significant difference in the frequency of MVPA reported retrospectively compared to that reported via mEPAs.
3. **Line 508:**
   - Social desirability bias is the propensity to self-report behaviour in a way that is viewed as desirable by society.
   - **Correction:** Social desirability bias refers to the tendency to self-report behavior in a manner perceived as socially acceptable.
4. **Line 516:**
   - Near real-time data collection minimizes the potential distortion in data reporting that may arise from retrospective recall bias.
   - **Correction:** Near-real-time data collection reduces the potential distortions in data reporting caused by retrospective recall bias.
5. **Line 568:**

- This association may be attributable to youth perceiving daily PA encouragement as overly pressuring or controlling, leading to resistance of MVPA.
- **Correction:** This association may be due to youth perceiving daily PA encouragement as overly pressuring or controlling, resulting in resistance to MVPA.

6. **Line 579:**
    - In particular, attending a school that was greater than five kilometres away from home was associated with a higher frequency of MVPA compared to those whose schools were within five kilometers from home.
    - **Correction:** Specifically, attending a school more than five kilometers from home was associated with a higher frequency of MVPA compared to attending a school within five kilometers from home.
7. **Line 601:**
    - Our study addresses critical gap by examining the differences in MVPA reporting via retrospective and mEPAs within the same cohort of individuals.

    - **Correction:** Our study addresses a critical gap by examining the differences in MVPA reporting via retrospective surveys and mEPAs within the same cohort of participants

Strengths:

1. **Innovative Use of Digital Technology:**
    - The use of ubiquitous devices like smartphones enhances participant engagement and facilitates scalable data collection.
2. **Ethical Digital Citizen Science Approach:**
    - The focus on participant autonomy and data privacy is a commendable aspect of the study design.
3. **Scalable Digital Epidemiology:**
    - The ability to recruit and engage participants remotely demonstrates the potential of digital tools for large-scale public health research.

Limitations:

1. **Design Limitations: Cross-Sectional with mEPAs:**
    - Future studies should consider adopting a longitudinal design to better assess the causal relationships and temporal changes in MVPA behavior.
2. **High Attrition Rate:**
    - Reduce the burden on participants by simplifying data collection protocols. Focusing solely on MVPA data could help improve participant compliance and retention.
3. **Limited Data Collection Period:**
    - Extend the prospective data collection period in future studies to capture a more comprehensive picture of MVPA behavior. Consider using longer timeframes to better assess habitual activity patterns.
4. **Validity and Reliability Concerns:**
    - Future studies should conduct validation tests for the modified survey to ensure it accurately measures MVPA frequency. Incorporating objective measures

(e.g., accelerometers, pedometers) could also enhance the reliability of the findings.

5. **Generalizability Issues:**
   - Increase the sample size and include diverse geographical locations in future studies to improve the external validity of the results. Adopting a multi-jurisdictional approach could help capture variations in MVPA behavior across different contexts.

Grammatical Corrections

1. **Line 604:**
   - This study adds novel evidence to our understanding of MVPA frequency among youth using advanced digital technologies.
   - **Correction:** This study provides novel evidence on the frequency of MVPA among youth using advanced digital technologies.
2. **Line 608:**
   - Limitations of this study include combining a cross-sectional design with mEPA measures, leading to the loss of important data points...
   - **Correction:** The limitations of this study include combining a cross-sectional design with mEPA measures, which led to the loss of important data points...
3. **Line 613:**
   - In this study a significant proportion of youth (85.75%) did not provide complete data for prospective measures...
   - **Correction:** In this study, a significant proportion of youth (85.75%) did not provide complete data for the prospective measures...
4. **Line 616:**
   - Participants were engaged prospectively using mEPAs across only eight days to enhance comparability with retrospective surveys.
   - **Correction:** Participants were engaged prospectively using mEPAs for only eight days to enhance comparability with the retrospective surveys.
5. **Line 622:**
   - Future research endeavour should be carried out in multiple jurisdictions...
   - **Correction:** Future research endeavors should be conducted in multiple jurisdictions...
6. **Line 627:**
   - The number of participants included in the analyses was lower due to participants not providing complete data throughout the study period.
   - **Correction:** The number of participants included in the analysis was reduced due to incomplete data provided by participants during the study period.
7. **Line 637:**
   - This study highlights the important differences in using retrospective vs. mEPAs measures, findings which are useful to develop appropriate prevention and management strategies for NCDs.
   - **Correction:** This study highlights the key differences between using retrospective and mEPA measures, findings that can inform the development of effective prevention and management strategies for NCDs.

Reference section:

- **Inconsistent Formatting:**

    - There are inconsistencies in the formatting of references, particularly regarding capitalization, punctuation, and the use of italics. For example, journal titles like "Progress in Cardiovascular Diseases" (Line 691) are not consistently italicized, and some references lack proper title case.

- **Missing Access Dates for Online Sources:**

    - Some references that include URLs (e.g., Lines 686 and 713) lack access dates. Including access dates for online sources is important for transparency and to indicate when the information was retrieved.

- **Redundant Citations:**
    - There are redundant citations of the same source, particularly for studies by Katapally TR (e.g., Lines 754, 795, and 829). These could be combined or referenced more efficiently to avoid repetition.
- **Citation Order:**
    - The references are not consistently ordered in the correct sequence based on their appearance in the text. For example, reference 51 (Line 830) appears before references 39 and 40 (Lines 795 and 799). Ensuring the correct order of citations is essential for coherence.
- **Incomplete References:**
    - A few citations lack complete information. For instance, Line 998 cites Van Domelen DR's study but does not provide a DOI or complete publication details. This could hinder readers from locating the source.
