# Supplementary material for: Digital citizen science for ethical monitoring of youth physical activity frequency: Comparing mobile ecological prospective assessments and retrospective recall
Source: PLOS Digit Health. 2025 May 2;4(5):e0000840. doi: 10.1371/journal.pdig.0000840 (PMC12047779; doi:10.1371/journal.pdig.0000840)
Supplement: Attachment — Submitted filename: Reviewer Responses.docx [file pdig.0000840.s003.docx]

**Reviewer 1**

1. The participants are representative of very small subset of youths and the study cannot be generalized upon youths in general.

Thank you for the suggestion. We acknowledge that the participants are representative of a small subset of youth. We have now made modifications to several sections of our manuscript to make this clearer. For instance:

a. **Abstract**: “However, these findings should be interpreted with caution, as the sample represents a small subset of youth, limiting generalizability to other youth populations.” (lines 52 to 53)
b. **Conclusion**: “These findings should be interpreted within the context of the study's limited sample representation. Further research is needed to assess the difference between retrospective and mEPA measures across diverse youth populations and settings.” (lines 649 to 651).

2. Only 68 people participated; and by the authors own comment- 273 participants are required for some level of statistical significance. Furthermore- these were selected via convenience sampling which is inherently non-random and not representative of the general population.

Thank you for your feedback. The sample size of 68 participants in this analysis is smaller than the originally planned 273 from the Smart Platform study, which was designed to achieve statistical significance for a broader set of variables (209 items). Since this analysis focused on specific variables, a smaller sample size was required to detect a statistically significant association, and we conducted a new sample size calculation a priori to reflect this narrower focus. We have now replaced this sample size calculation in the statistical analysis for clarification: “A priori power analysis determined that a minimum sample size of 59 participants was required, based on an effect size of 0.15 and a power of 90%, which was lower than the number of participants who provided complete data in this study.” (lines 342 to 345).

Nevertheless, we acknowledge that a large proportion of youth dropped out from the study, which can negatively impact the representative of our sample. Thus, we enhanced our discussion of our comparison of the baseline demographics in the original sample (N=436) versus the final sample with complete data for our analysis (N=68). We have now also added to the Limitations: “A significant proportion of youth (85.75%) did not provide complete data for prospective measures, reducing the final sample size and limiting the ability to conduct subgroup analyses, including more nuanced ethnic categories. Nevertheless, the baseline demographics of those with complete data did not differ significantly from the original sample, suggesting that external validity was not substantially compromised” (lines 607 to 612).

Regarding the comment about convenience sampling, we have now added to the Limitations: “Additionally, as convenience sampling does not produce a random or representative sample of the general population, incorporating random or stratified sampling methods in future research could improve the robustness and generalizability of results.” (lines 636 to 638).

3. All participants are from the same city in Canada. In the absence of specifying which schools this was conducted over- it’s unclear whether participants are from the socio-economic or (Catholic) religious background

Thank you for your feedback. We have now added to the Methods: “School affiliation was accounted for as a categorical variable (1–5) representing the different schools included in the study.” (lines 309 to 311). However, to maintain participant confidentiality, we did not disclose specific school names. This approach ensures that potential socio-economic and religious differences between schools are considered in the analysis while protecting participant privacy. Additionally, we adjusted for parental education, which is highly correlated with household income and has been used in previous literature

1) <https://pubmed.ncbi.nlm.nih.gov/29162183/>
2) <https://www.nature.com/articles/nn.3983>
4. The study appears to be conducted at fixed point during the academic cycle. This date range is not provided; and there is no discussion on seasonality (e.g.- students are less likely to be active over winter vs. Spring)

The primary goal of this study was to compare physical activity frequency between different participant groups, both retrospectively and prospectively. The study was conducted over a four-month period (August 31st to December 31st) (lines 39 to 40; lines 175 to 176), with data for each group collected within a short, consecutive 8-day period. Additionally, because data collection within each school occurred during a similar timeframe, any potential seasonality effects were minimized. Furthermore, by controlling for school in our analysis, we accounted for any school-specific seasonal influences, reducing the likelihood that seasonal variations in physical activity affected the results.

Nevertheless, we have enhanced the Discussion section by adding to lines 496 to 502:
However, these associations may be influenced by unmeasured seasonal and weather-related variations in activity patterns [75,76]. Fluctuations in temperature, daylight hours, and other environmental factors such as air quality are important determinants of active transportation and overall MVPA, particularly in regions with distinct seasonal changes [77–79]. Additionally, methodological advancements using cross-sectional accelerometry data have demonstrated the role of weather and seasonality on active living behaviours [76]. Future studies should integrate seasonality and objective weather measures to refine MVPA models and improve accuracy.”

5. For some surveyed data- “Don’t know” isn’t provided as an option; for instance- when students may not know how far they live away from their schools

Thank you for your feedback. We have now added to the Discussion: “Measurement limitations may have also influenced findings, as the absence of an "I do not know" option in some survey questions, such as school distance, could have led to incomplete or inaccurate responses. Refining survey design in future research can enhance data accuracy and better capture contextual factors associated with MVPA.” (lines 563 to to 567).

6. The study has had a very high attrition rate (>85%)- I’d like to see this discussed in greater length. Were there any systematic differences between those who completed the study and those who dropped out? This could significantly bias the results.

Thank you for your feedback. We have stated in the results: “There were no significant differences between the baseline and final sample across all included variables, except for school (p = 0.008).” (lines 384 to 386). We have added to the Abstract: “However, findings should be interpreted with caution, as the sample represents a small subset of youth, limiting generalizability to other youth populations.” (lines 52 to 53) and Strengths and Limitations: “Nevertheless, the baseline demographics of those with complete data did not differ significantly from the original sample, suggesting that external validity was not substantially compromised.” (lines 610 to 613)

We have also added to the Limitations: “While the digital epidemiological approach enabled large-scale recruitment and real-time engagement, it may have also placed a burden on participants. A significant proportion of youth (85.75%) did not provide complete data for prospective measures, reducing the final sample size and limiting the ability to conduct subgroup analyses, including more nuanced ethnic categories. Nevertheless, the baseline demographics of those with complete data did not differ significantly from the original sample, suggesting that external validity was not substantially compromised. As a result of the smaller sample size, only a few covariates were included in the statistical models to minimize overfitting, which could c­­­ontribute to residual confounding. This limitation is further compounded by the short study duration (8 days), which restricts the ability to capture longer-term MVPA patterns and potential fluctuations due to external factors such as school schedules, weather changes, or social influences [75,78,95]. Future studies could benefit from extending the data collection period or conducting a longitudinal cohort study (>1 year) to improve statistical power and allow for stronger conclusions about causality and directionality. Additionally, randomized controlled trials could be used to evaluate the effectiveness of mEPAs in promoting physical activity, assessing whether real-time monitoring and feedback mechanisms lead to sustained behavioural changes. Leveraging performance-based incremental incentives for completing mEPA prompts could also enhance engagement and reduce attrition rates in future MVPA studies [108]." (lines 606 to 625).

7. The study should call out whether participants were aware of the participation kickback ahead of time- as the $25 YMCA pass is likely to attract/ retain a demographic of students that is inherently very different

Thank you for your feedback. We have now added this to the Methods (lines 201 to 207): “All youth were provided with a one-week free pass to the local YMCA, valued at $25, through a partnership between our research team and the YMCA [51]. The pass was intended to provide participants with access to community-based physical activity resources during the study period. To avoid coercion, participants were informed that accepting the pass was optional and that their decision to participate or withdraw from the study would not affect their eligibility for the pass. Ultimately, fewer than 5% of participants used the pass, suggesting it had minimal impact on participation decisions or demographic representation [34].”.

8. The ethnicities list does not include common demographic groups like Asians, Blacks, etc.

Thank you for your feedback. The ethnicities list in our study was designed to reflect the diversity of the population in Saskatchewan, where the study was conducted. Categories such as “African” and “Asian” were included to capture ethnic groups based on cultural or regional backgrounds. We understand that "Black" is often used as a racial category, but since the focus of our study was on ethnicity, we chose to use labels such as “African” and “Asian” to capture ethnic backgrounds linked to specific regions.

Moreover, due to the low number of responses within individual ethnic categories, we grouped several responses under the "Visible minorities" category, which included “African,” “Asian,” “Caribbean/West Indian,” “Eastern European,” “European,” and “South Asian” to ensure sufficient sample size for analysis. This grouping limited the ability to perform detailed comparisons within each ethnic group. We have now added to the Limitations: “A significant proportion of youth (85.75%) did not provide complete data for prospective measures, reducing the final sample size and limiting the ability to conduct subgroup analyses, including more nuanced ethnic categories. […] Future studies could benefit from extending the data collection period or conducting a longitudinal cohort study (>1 year) to improve statistical power and allow for stronger conclusions about causality and directionality. Additionally, randomized controlled trials could be used to evaluate the effectiveness of mEPAs in promoting physical activity, assessing whether real-time monitoring and feedback mechanisms lead to sustained behavioural changes. Leveraging performance-based incremental incentives for completing mEPA prompts could also enhance engagement and reduce attrition rates in future MVPA studies [108]. (lines 607 to 625).

9. The actual survey conducted isn’t provided in the figshare link. Also, the author stated that the survey was adapted from three validated questionnaires but need to provide more information about the adaptation process and how they ensured the validity and reliability. Did they conduct any pilot testing or cognitive interviews to assess the clarity and comprehensibility of the questions?

Thank you for your feedback. We have now shared the survey conducted in the figshare link.

As the study utilized a digital citizen science approach, the surveys were deployed via a custom-built application, enabling real-time feedback from youth through their smartphones. We have now clarified in the Methods: “As this study utilized a digital citizen science approach, youth could communicate with researchers in real time via the app to provide feedback on data collection strategies, including prompt timing and the comprehensibility and clarity of survey questions.” (lines 169 to 172).

Additionally, our adaptation process required us to modify the original paper-based surveys for digital administration. While the original surveys were validated, pilot testing or validation specific to the digital format was not conducted in this study. Instead, we focused on adapting the surveys to be compatible with the digital platform and ensuring consistency between retrospective and prospective measures. However, we acknowledge the importance of validating these measures for digital administration to ensure they retain their validity and reliability. We have now added to the Limitations: “The modified survey was based on three validated surveys [3,44,45]; however, both retrospective and prospective measures assume typical MVPA habits, which may limit generalizability. Another limitation is that adapting validated surveys into a digital format required modifications that could introduce measurement error due to differences in user interface and response burden [20,21]. Future research should validate these measures for digital administration and refine survey designs to include response options for uncertainty. Integrating objective data collection methods, such as accelerometers and pedometers, could further enhance MVPA measurement accuracy [75,109,110].” (lines 628 to 638).

10. The authors need to provide more information on the specific timing and frequency cadence of the mEPA prompts. How many prompts were sent per day and when? Was the timing randomized or fixed?

Thank you for your suggestion. Only one prompt was sent per day and the timing was fixed. We have now added content to the Methods to clarify the timing and frequency of the mEPA prompts: “The mEPA prompts were sent once a day at a fixed time, specifically between 8:00 PM and 11:30 PM and could be completed before 12am (midnight). The timing was fixed rather than randomized to align with typical end-of-day reflection and to reduce variability in reporting windows” (lines 234 to 237).

11. The abstract is very long e.g.- third sentence is 64 words long and should be split to make it more readable. The abstract should also be restructured such that it is more concise and engaging and has the study findings nearer to the start of the abstract. The abstract should also call out why this study is important and what the key findings are.

Thank you for your feedback. We have now cut down the abstract to 247 words and followed a structured format to make it more concise and engaging. We have added a section for the importance of the study (lines 31 to 35), objective of the study (lines 36 to 38), key findings (lines 45 to 48), and implications (lines 49 to 53).

12. The study’s goal is to compare data gathered from smartphone surveys vs. time triggered assessments. The author summary calls out in greater detail how these two are different.

Thank you for your feedback. In response to your comment, we have revised the abstract to emphasize the distinction between the two data collection methods used in the study: retrospective surveys and prospective mobile ecological momentary assessments (mEPAs). Specifically, the abstract now states: “Significant differences were found in the frequency of MVPA reported via retrospective surveys versus mEPAs (p < 0.000). Prospective MVPA frequency was associated with family and friend support, having drug-free friends, part-time employment, and school distance. Retrospective MVPA frequency was significantly associated with school and strength training." (lines 45 to 48). We hope this revision addresses your concern and makes the abstract clearer.

13. The paper feels like it could be significantly trimmed down. E.g.- L222-228. This is not relevant to the study and should be removed.

Thank you for your feedback. We have now trimmed down a significant amount of content

from the manuscript to improve concision. We cut down the Abstract to 250 words and

the Introduction from 1056 words to 656 words. We also trimmed the content regarding

citizen science based on the lines you suggested in the Methods. However, to address

the other reviewer comments we had to add more content to the Methods and Discussion,

which added to the length of the study. Nevertheless, we aimed to address all feedback in

a concise manner to trim down the paper as suggested.

14. Some sentences need to be checked; e.g.- “Youth were predominantly males” isn’t true if looking at the accompanying table.

Thank you for point this out. We have now thoroughly reviewed the manuscript to ensure consistency. We have modified the statement regarding sociodemographic characteristics to make the results clearer: The participants were predominantly female (56.72%), with 40.30% identifying as male, and 2.98% identifying as transgender, other, or preferring not to disclose.” (lines 376 to 377).

15. Explain why the specific age range of 13-21 years was chosen vs. a tighter band like 18-29.

Thank you for your question. We have now added to the Methods: “The age range of 13-21 years was chosen to capture a broad spectrum of youth during key developmental stages, from adolescence into early adulthood [41,42].” (lines 176 to 178). This range includes individuals who are transitioning through important stages of physical, social, and behavioral development. A tighter band like 18-29 years would exclude adolescents, missing out on a critical period of growth and activity patterns. Additionally, the broader range helps provide more comprehensive data on youth physical activity across a variety of life stages. Nevertheless, age was controlled for in the analysis to ensure that this broader range did not negatively impact or confound the results.

16. Clarify the rationale for providing a YMCA pass to participants.

Thank you for your feedback. We have now added to the Methods: “All youth were provided with a one-week free pass to the local YMCA, valued at $25, through a partnership between our research team and the YMCA [51]. The pass was intended to provide participants with access to community-based physical activity resources during the study period. To avoid coercion, participants were informed that accepting the pass was optional and that their decision to participate or withdraw from the study would not affect their eligibility for the pass. Ultimately, fewer than 5% of participants used the pass, suggesting it had minimal impact on participation decisions or demographic representation.” (lines 201 to 207).

**Reviewer 2**

1. The title: I recommend the title: “Digital Citizen Science for Ethical Monitoring of Youth Physical Activity: Comparing Mobile Ecological Prospective Assessments and Retrospective Recall.” Consider capitalizing the start letter for every word in the title.

Thank you for your recommendation. We have improved the title of the manuscript and capitalized the start letters “Digital Citizen Science for Ethical Monitoring of Youth Physical Activity Frequency: Comparing Mobile Ecological Prospective Assessments and Retrospective Recall”.

Introduction

1. The rationale for comparing retrospective recall with mobile ecological prospective assessments (mEPAs) is only briefly mentioned. The introduction would benefit from a clearer justification for why this comparison is crucial, especially in a youth cohort. I recommend Strengthening the rationale by providing a more detailed explanation of the limitations of retrospective recall, particularly in youth populations, and the potential advantages of using MEPs.

Thank you for your recommendation. We have now provided a more focused analysis of the advantages and limitations of mEPAs in comparison to retrospective recall, particularly among youth

We have refined our discussion on the limitations of retrospective recall on lines x to x: “Traditional retrospective self-report measures, commonly used in MVPA surveillance, are prone to recall biases, social desirability effects, and difficulty in tracking daily activity patterns [12,13]. These limitations highlight the need for more accurate and ecologically valid measurement approaches, particularly for youth whose PA behaviours fluctuate depending on social and environmental contexts [14,15] (lines 94 to 98).

We have also concisely stated the direct benefits of mEPAs compared to retrospective recall: “Compared to retrospective measures, mEPAs offer structured end-of-day reflections that may improve recall for structured and scheduled activities, such as organized sports or social PA events, while reducing participant burden [22].” (lines 109 to 111).

Finally, we ground this discussion of comparing mEPAs and retrospective recall in SCT theory when stating our hypothesis on lines x to x: “Given the recall biases and self-report limitations of retrospective measures [31], we hypothesize that mEPAs, which provide structured, end-of-day reflections, will yield different MVPA estimates by encouraging self-regulation and reinforcing goal-setting – key components of SCT [23,27].” (lines 130 to 133).

2. The transition from conventional ecological momentary assessments to the adapted mEPAs is somewhat abrupt. The decision to modify the reporting interval is explained, but there is limited discussion on the potential trade-offs of this change (e.g., reduced real-time accuracy). You can provide a more balanced discussion of the benefits and limitations of adapting momentary assessments to end-of-day reporting, including potential impacts on data quality.

Thank you for your feedback. We have now provided a more balanced discussion on

lines 104 to 111: “However, mEMAs require frequent prompts throughout the day, which can be disruptive for youth balancing academic and social commitments [19]. The high response burden can lead to participant fatigue, which limits the feasibility of mEMAs in youth populations [20,21]. To address these challenges, we adapted mEMAs by shifting the reporting interval to the end of the day, a method referred to as mobile Ecological Prospective Assessments (mEPAs). Compared to retrospective measures, mEPAs offer structured end-of-day reflections that may improve recall for structured and scheduled activities, such as organized sports or social PA events, while reducing participant burden [22].”

We continued this discussion when introducing our theoretical framework: “While real-time assessments like mEMAs can capture these dynamic influences, they may not fully account for self-regulation and reflection, which are critical for sustained PA engagement [23,27]. By integrating structured opportunities for self-reflection, mEPAs reinforce critical SCT mechanisms, such as goal-setting and self-monitoring, helping youth consolidate PA experiences, recognize behaviour patterns, and adjust their future actions accordingly [27].” (lines 116 to 121).

3. The use of Social Cognitive Theory is mentioned briefly without much detail. The connection between the theory and the research design could be elaborated to demonstrate how it informs the choice of assessment methods (retrospective vs. mEPAs). You must expand on the application of Social Cognitive Theory and its relevance to the selection of assessment tools and measurement of PA among youth.

Thank you for your feedback. We have expanded on Social Cognitive Theory (SCT) to

provide a more detailed explanation of how it informed our choice of assessment methods

(lines x to x): " Social Cognitive Theory (SCT) provides a useful framework for evaluating PA measurement approaches by emphasizing the interaction of personal, environmental, and social factors on behaviour [23,24]. Youth PA engagement is shaped by peer interactions, parental support, and access to recreational spaces – factors that vary throughout the day [25,26]. While real-time assessments like mEMAs can capture these dynamic influences, they may not fully account for self-regulation and reflection, which are critical for sustained PA engagement [23,27]. By integrating structured opportunities for self-reflection, mEPAs reinforce critical SCT mechanisms, such as goal-setting and self-monitoring, helping youth consolidate PA experiences, recognize behaviour patterns, and adjust their future actions accordingly [27].” (lines 113 to 121).

We also reintroduced the theory in our hypothesis statement: “Given the recall biases and self-report limitations of retrospective measures [48], we hypothesize that mEPAs, which provide structured, end-of-day reflections, will yield different MVPA estimates by encouraging self-regulation and reinforcing goal-setting – key components of SCT.” (lines 130 to 133).

This expansion also allowed us to highlight the advantages of mEPAs over mEMAs by emphasizing their alignment with self-regulatory processes and structured reflection, as outlined in SCT.

4. The challenges related to digital literacy differences, device access, and compliance with digital assessments are not adequately addressed. I think it’s good to discuss potential barriers to implementing digital citizen science approaches, such as disparities in access to smartphones or varying levels of digital literacy among participants.

Thank you for your feedback. We further discusses the challenges related to digital literacy, device ownership, and digital assessment compliance on: “The feasibility of implementing prospective digital assessments at scale may be influenced by disparities in smartphone access and internet connectivity, which could limit participation and introduce selection bias [104–106]. Future research should examine how differences in technology access impact participation and data quality. Additionally, exploring alternative delivery methods, such as hybrid digital and low-bandwidth approaches could help improve accessibility and ensure broader participation in digital PA monitoring [106]. In addition to access challenges, despite increasing digital literacy levels overall, participants with lower digital literacy may struggle with app navigation, leading to missing or inconsistent data [106,107]. Providing user-friendly interfaces and technical support could help mitigate these issues.” (lines 587 to 596).

5. The hypothesis is not fully integrated with the theoretical framework or the limitations of previous research. Additionally, the study aim could be more concisely stated. You can link the hypothesis to the identified gaps in the literature and provide a concise and focused statement of the study’s objectives.

Thank you for your feedback. We have now strengthened the integration of our hypothesis with the theoretical framework and previous research limitations (lines 130 to 133):

“While several studies have compared retrospective and momentary assessments of human behaviour, including sedentary behaviour [28], PA [29], and substance use [30], in adult populations, limited research has examined how youth engage with smartphones to report MVPA using both digitally deployed retrospective surveys and mEPAs. Thus, this study aims to engage youth as citizen scientists [32] to compare MVPA frequency reported via retrospective surveys and mEPAs within the same cohort. Additionally, it accounts for sociodemographic, behavioural, and contextual factors that may influence reporting differences, allowing for a more controlled comparison between methods. Given the recall biases and self-report limitations of retrospective measures [31], we hypothesize that mEPAs, which provide structured, end-of-day reflections, will yield different MVPA estimates by encouraging self-regulation and reinforcing goal-setting – key components of SCT [23,27].”.

Methodology

1. Study Design: I recommend to Provide a brief discussion on the potential ethical issues related to digital surveillance and consent, especially for minors. Clarify how data privacy is maintained in the Smart Platform.

Thank you we have further strengthened the Data and risk management section by adding how data privacy was maintained within the Smart Platform (Lines 324 to 337):
The app used in this study was designed to prioritize confidentiality, data privacy, safety, and security. All data were encrypted before being transmitted to a secure cloud server, and access to personally identifiable information on participants' smartphones (e.g., site visits, location, and contact lists) was restricted. A multi-layered security approach was implemented, including robust encryption [49] to protect against eavesdropping, man-in-the-middle attacks, and rogue hotspots [50]. To enhance control over data sharing, participants could use the “snooze” function to pause transmission. Additionally, youth data were encrypted using a hash algorithm, with MAC address anonymization, ensuring that device identifiers were irreversibly transformed to protect participant privacy. During in-person recruitment, participants were informed about privacy protections, the option to opt out, and the ability to control data uploads [34,35]. They could choose to upload data only when connected to Wi-Fi or when their devices were plugged into a power source. Clear instructions on withdrawing from the study were provided within the app, and participants could contact the research team through dedicated Smart Platform study emails for support and inquiries.”.

2. Participants and Recruitment: I recommend to : Acknowledge the limitations of using convenience sampling.

Thank you for your recommendation. We have now added to Limitations: “Additionally, as convenience sampling does not produce a random or representative sample of the general population, incorporating random or stratified sampling methods in future research could improve the robustness and generalizability of results.” (lines 636 to 638).

3. Provide more details on how implied consent was obtained and any steps taken to ensure ethical compliance.

Thank you for providing the opportunity to further clarify our implied consent process. We have now updated the participant section with the implied informed consent information:

“Informed consent was provided by all youth who participated in the study through the app. Implied informed consent was obtained from parents and caregivers of participants aged 13 and 16 years old. Before in-person recruitment sessions, parents were provided the opportunity to read about the study, ask questions, and contact the research team if they did not want their child to participate. To facilitate this process, we collaborated with school administrators who emailed the informed consent forms to each household before the scheduled recruitment sessions. Parents or caregivers were required to review, sign, and provide explicit agreement for their child’s participation. If we did not receive a signed consent form, we understood this as a lack of permission, indicating that the parent or caregiver did not wish for their child to participate. This approach ensured that participation was entirely voluntary, and that parental consent was obtained before involving any participant in the study.” (Lines 189 to 199).

4. Measures and Data Collection: I recommend mentioning the potential biases in self- reported measures and justify using end-of-day reporting for mEPAs as a strategy to mitigate compliance issues.

Thank you for your recommendation. In response to other reviewer comments, we have now mentioned the biases associated with self-reported measures and provided a justification of mEPAs as a strategy to mitigate compliance issues in the Introduction:

“Traditional retrospective self-report measures, commonly used in MVPA surveillance, are prone to recall biases, social desirability effects, and difficulty in tracking daily activity patterns [12,13]. These limitations highlight the need for more accurate and ecologically valid measurement approaches, particularly for youth whose PA behaviours fluctuate depending on social and environmental contexts [14,15].” […] However, mEMAs require frequent prompts throughout the day, which can be disruptive for youth balancing academic and social commitments [19]. The high response burden can lead to participant fatigue, which limits the feasibility of mEMAs in youth populations [20,21]. To address these challenges, we adapted mEMAs by shifting the reporting interval to the end of the day, a method referred to as mobile Ecological Prospective Assessments (mEPAs). Compared to retrospective measures, mEPAs offer structured end-of-day reflections that may improve recall for structured and scheduled activities, such as organized sports or social PA events, while reducing participant burden [22]. (lines 91 to 111).

5. Ethical Considerations: I recommend to include a brief note on how incentives were balanced with ethical considerations to avoid coercion.

Thank you for your recommendation. “All youth were provided with a one-week free pass to the local YMCA, valued at $25, through a partnership between our research team and the YMCA [51]. The pass was intended to provide participants with access to community-based physical activity resources during the study period. To avoid coercion, participants were informed that accepting the pass was optional and that their decision to participate or withdraw from the study would not affect their eligibility for the pass. The incentive was introduced at the start of the study to ensure participants had full knowledge of it before providing consent, allowing them to make an informed decision without undue influence. Ultimately, fewer than 5% of participants used the pass, suggesting it had minimal impact on participation decisions or demographic representation.” (lines 201 to 207).

6. Discuss any measures taken to ensure participants were aware of their rights and data usage.

Thank you for your suggestion. We have now added to lines 324 to 337: “The app used in this study was designed to prioritize confidentiality, data privacy, safety, and security. All data were encrypted before being transmitted to a secure cloud server, and access to personally identifiable information on participants' smartphones (e.g., site visits, location, and contact lists) was restricted. A multi-layered security approach was implemented, including robust encryption [49] to protect against eavesdropping, man-in-the-middle attacks, and rogue hotspots [50]. To enhance control over data sharing, participants could use the “snooze” function to pause transmission. Additionally, youth data were encrypted using a hash algorithm, with MAC address anonymization, ensuring that device identifiers were irreversibly transformed to protect participant privacy. During in-person recruitment, participants were informed about privacy protections, the option to opt out, and the ability to control data uploads [34,35]. They could choose to upload data only when connected to Wi-Fi or when their devices were plugged into a power source. Clear instructions on withdrawing from the study were provided within the app, and participants could contact the research team through dedicated Smart Platform study emails for support and inquiries [34,35].”.

7. Measurement of Independent Variables: Recommendation: Consider explaining the rationale for dichotomizing these variables. A sensitivity analysis could be conducted to evaluate the impact of this decision on the study outcomes.

Thank you for the opportunity to rationalize dichotomizing the variables: distance from home to school, strength training and peer support for MVPA. We have included a rationale for each of the variables in the measures section: Distance from home to school “…This dichotomization was necessary to distinguish between distances that are feasibly covered by active modes of transport (e.g., walking or cycling) and those that typically necessitate motorized transport. Also, sample distribution revealed a clustering, with a higher frequency of participants residing within 5 km of school, which supports this dichotomization…” (Lines 281 to 285) Strength training “…This dichotomization is based on evidence suggesting that even one session per week can improve strength and help offset age-related declines [48] …” (Lines 319 to 321), and peer support of MVPA “… This dichotomization was implemented to differentiate participants with no peer support from those with any level of peer influence …” (Lines 258 to 259). We believe that given the study’s objectives and the natural distribution of responses, this categorization provides meaningful insights while minimizing the risk of small subgroup sizes that could compromise statistical reliability.

8. Data Collection and Risk Management: Recommendation: Provide additional details on the encryption method used and discuss potential risks associated with data transfer, especially over public Wi-Fi.

Thank you for your recommendation. We have now added all details requested to the Data and Risk Management section (lines 324 to 337).

9. Inclusion Criteria and Data Analyses: Recommendation: Clarify the method used for handling missing data (e.g., listwise deletion, imputation) and provide a rationale for the choice of Poisson regression, including any steps taken to check for overdispersion.

Thank you. We have written a sentence on how we handled missing data: “Given the high proportion of missing MVPA data, particularly in the mEPA measures, a complete case analysis was used to minimize potential bias from imputation. While this reduced the sample size, it allowed for direct comparisons between retrospective and mEPA MVPA measures.”. (Lines 345 to 348).

We have also changed our analysis technique to Negative binomial regression: “Negative binomial regression was used to assess factors associated with retrospective and mEPA frequencies. The overdispersion parameter from the Poisson regression analysis (Retrospective: 1.399; mEPA: 1.081) indicated that the data were overdispersed, making negative binomial regression a more appropriate model.” (Lines 354 to 358)

10. Covariate Selection: Recommendation: Provide a brief explanation of why these specific covariates were chosen and discuss any limitations related to unmeasured confounders.

Thank you for your feedback. We have now added to the Methods:

“Covariates, including peer and family support [25,52], strength training [53,54], distance and friends' commitment to being drug-free, were selected based on their established associations with physical activity engagement. Peer and family support align with SCT by shaping self-efficacy and providing vicarious reinforcement, which can motivate youth to engage in MVPA [25,52]. Social factors, such as proximity to active peers and substance-free environments, have also been linked to participation in structured and recreational physical activities, aligning with SCT’s emphasis on environmental determinants of behaviour. Strength training was included given its role in overall MVPA patterns and its increasing emphasis in youth exercise guidelines [53,54], though it is not explicitly part of SCT.” (lines 360 to 368).

We also added to the Limitations: “As a result of the smaller sample size, only a few covariates were included in the statistical models to minimize overfitting, which could c­­­ontribute to residual confounding. This limitation is further compounded by the short study duration (8 days), which restricts the ability to capture longer-term MVPA patterns and potential fluctuations due to external factors such as school schedules, weather changes, or social influences [75,78,95]. Future studies could benefit from extending the data collection period or conducting a longitudinal cohort study (>1 year) to improve statistical power and allow for stronger conclusions about causality and directionality. Additionally, randomized controlled trials could be used to evaluate the effectiveness of mEPAs in promoting physical activity, assessing whether real-time monitoring and feedback mechanisms lead to sustained behavioural changes. Leveraging performance-based incremental incentives for completing mEPA prompts could also enhance engagement and reduce attrition rates in future MVPA studies [108]. (lines 614 to 625).

Results

1. Sample Size and Data Completeness: Try to: Discuss the implications of the reduced sample size on the study's external validity. Consider addressing any potential biases introduced by excluding participants with incomplete data.

Thank you for your feedback. We have conducted analyses to assess the difference in baseline demographics across the original sample and the complete data sample and found that only one out of the 11 sociodemographic variables was significantly different across the two samples. We have now added to the Results: “There were no significant differences between the baseline and final sample across all included variables, except for school (p = 0.008).”. (lines 384 to 386).

Additionally, we have now added to the Limitations: “While the digital epidemiological approach enabled large-scale recruitment and real-time engagement, it may have also placed a burden on participants. A significant proportion of youth (85.75%) did not provide complete data for prospective measures, reducing the final sample size and limiting the ability to conduct subgroup analyses, including more nuanced ethnic categories. Nevertheless, the baseline demographics of those with complete data did not differ significantly from the original sample, suggesting that external validity was not substantially compromised. As a result of the smaller sample size, only a few covariates were included in the statistical models to minimize overfitting, which could contribute to residual confounding. This limitation is further compounded by the short study duration (8 days), which restricts the ability to capture longer-term MVPA patterns and potential fluctuations due to external factors such as school schedules, weather changes, or social influences [75,78,95]. Future studies could benefit from extending the data collection period or conducting a longitudinal cohort study (>1 year) to improve statistical power and allow for stronger conclusions about causality and directionality. Additionally, randomized controlled trials could be used to evaluate the effectiveness of mEPAs in promoting physical activity, assessing whether real-time monitoring and feedback mechanisms lead to sustained behavioural changes. Leveraging performance-based incremental incentives for completing mEPA prompts could also enhance engagement and reduce attrition rates in future MVPA studies [108].” (lines 606 to 625).

2. Summary Statistics and Descriptive Analysis: I suggest including a comparison of key demographics between the initial and final samples to evaluate representativeness and potential selection bias.

Thank you for your feedback. The comparison of key demographics between initial and final samples is now presented in Table 1. We have also added to lines 384 to 386: “There were no significant differences between the baseline and final sample across all included variables, except for school (p = 0.008).”.

3. Outcome Variables and Statistical Tests: I suggest Reporting the effect size (e.g., Cohen's d or rank-biserial correlation) for the Wilcoxon signed-rank test to provide a better understanding of the magnitude of the difference.

Thank you for your suggestion. We have now added the Cohen’s d effect size to the

Results section for the Wilcoxon signed-rank test (Cohen’s d = 1.37 95% C.1 = 0.900,

1.839 - see Table 2.

4. Poisson Regression Analysis: So, I suggest addressing potential overdispersion by reporting the dispersion parameter and, if necessary, using a negative binomial regression model.

Thank you for your feedback. We have now added lines 354 to 358: “Negative binomial regression was used to assess factors associated with retrospective and mEPA frequencies. The overdispersion parameter from the Poisson regression analysis (Retrospective: 1.399; mEPA: 1.081) indicated that the data were overdispersed, making negative binomial regression a more appropriate model.”.

5. Provide more context and interpretation for unexpected findings, such as the negative association between daily family support and prospective MVPA and why youth with active friends reported lower MVPA.

Thank you for your feedback. We have now added content to the Discussion to enhance this explanation on lines 532 to 554.

6. Presentation of Results: Recommendation: Simplify the tables by using more evident labels and footnotes to explain abbreviations.

Thank you for your suggestion. We have simplified all tables with more evident labels and footnotes.

Discussion

1. Main Findings and Interpretation: Recommendation: Include a brief discussion on the practical implications of using mEPAs for MVPA monitoring in youth, especially in terms of informing public health guidelines.

Thank you for your recommendation. We have now added to the Discussion:

“From a public health perspective, mEPAs provide a structured and participant-driven approach to PA monitoring [36]. Unlike passive methods, which continuously collect information and raise privacy concerns, mEPAs require participants to actively report their PA, giving them greater control over what and when they share [33–35]. This makes mEPAs a feasible option for large-scale PA surveillance, particularly in youth populations where ethical considerations around digital tracking are heightened [46]. If implemented at scale, mEPAs could help policymakers and educators identify when and where youth are least active, leading to more targeted interventions [84]. For instance, if reports show lower PA levels on Mondays and Fridays, schools could introduce structured movement breaks to maintain consistency. Municipalities could also use mEPAs to assess whether recreation programs align with youth activity patterns [26]. If evening PA is low, extending operating hours at recreation centres or improving park lighting could encourage participation. (lines 574 to 585).

2. Address the limitations of both assessment methods, including potential biases and challenges related to participant compliance.

Thank you for providing the opportunity to further clarify the limitations of both assessment methods. We have now added to the Discussion: “The feasibility of implementing prospective digital assessments at scale may be influenced by disparities in smartphone access and internet connectivity, which could limit participation and introduce selection bias [104–106]. Future research should examine how differences in technology access impact participation and data quality. Additionally, exploring alternative delivery methods, such as hybrid digital and low-bandwidth approaches could help improve accessibility and ensure broader participation in digital PA monitoring [106]. In addition to access challenges, despite increasing digital literacy levels overall, participants with lower digital literacy may struggle with app navigation, leading to missing or inconsistent data [106,107]. Providing user-friendly interfaces and technical support could help mitigate these issues. Sustained participant engagement is another key consideration, as compliance with mEPAs may decline over time due to notification fatigue, competing priorities, and survey burden [20,21]. Research on optimizing adherence strategies, such as gamification, adaptive prompting, or incentives, could enhance long-term engagement in digital PA monitoring.” (lines 587 to 599).

3. Comparison with Previous Studies: Recommendation: Provide a more detailed explanation for the unexpected findings, such as the negative association between having active friends and MVPA frequency. Consider discussing potential underlying mechanisms, such as social comparison effects or differences in the sample population.

Thank you for your feedback. We have now added content to the Discussion to enhance this explanation on lines 532 to 554:

“On the contrary, our study interestingly found that youth reporting that an adult encouraged PA daily was associated with less frequency of MVPA. This finding may be explained by psychological reactance, where frequent encouragement is perceived as controlling rather than supportive, leading youth to resist PA as a way to assert autonomy [96]. Previous research has suggested that excessive parental involvement in PA can sometimes lead to negative motivational outcomes, particularly if the encouragement is perceived as pressure rather than genuine support [93,95,97]. Future studies could explore whether the tone, context, or delivery of PA encouragement influences youth engagement, as well as whether encouragement is more effective when it is self-directed rather than externally imposed.

In addition to family encouragement, our study found that having one or more physically active friends was associated with lower frequency of MVPA, which contrasts with previous studies conducted among youth [15]. One possible explanation for this unexpected finding is the role of social comparison. Some youth may compare their PA levels to those of their highly active peers, leading to feelings of inadequacy that reduce motivation to engage in PA themselves [98], making them feel inferior or less motivated to engage in MVPA themselves. This aligns with research on self-efficacy, which suggests that individuals who doubt their ability to match their peers' activity levels may be less likely to participate in PA. For instance, Schroeder et al. (2020) found that self-efficacy was associated with high MVPA levels and low sedentary behaviour among youth [99]. Future research should explore how social comparisons influence youth PA behaviours and whether interventions that build self-efficacy can mitigate these effects.”.

4. Theoretical and Practical Implications: Recommendation: Include concrete suggestions for policymakers, educators, and public health practitioners on how to utilize mEPAs for monitoring and promoting physical activity among youth.

Thank you for your recommendation. We have now added this content to lines 574 to 585: From a public health perspective, mEPAs provide a structured and participant-driven approach to PA monitoring [36]. Unlike passive methods, which continuously collect information and raise privacy concerns, mEPAs require participants to actively report their PA, giving them greater control over what and when they share [33–35]. This makes mEPAs a feasible option for large-scale PA surveillance, particularly in youth populations where ethical considerations around digital tracking are heightened [46]. If implemented at scale, mEPAs could help policymakers and educators identify when and where youth are least active, leading to more targeted interventions [84]. For instance, if reports show lower PA levels on Mondays and Fridays, schools could introduce structured movement breaks to maintain consistency. Municipalities could also use mEPAs to assess whether recreation programs align with youth activity patterns [26]. If evening PA is low, extending operating hours at recreation centres or improving park lighting could encourage participation.

5. Limitations of the Study: Recommendation: Clearly state the limitations related to the reduced sample size and potential selection bias. Discuss the challenges of using digital tools for data collection, especially among diverse youth populations with varying levels of access and digital skills.

Thank you for your feedback. We have now added to the Limitations: “While the digital epidemiological approach enabled large-scale recruitment and real-time engagement, it may have also placed a burden on participants. A significant proportion of youth (85.75%) did not provide complete data for prospective measures, reducing the final sample size and limiting the ability to conduct subgroup analyses, including more nuanced ethnic categories. Nevertheless, the baseline demographics of those with complete data did not differ significantly from the original sample, suggesting that external validity was not substantially compromised. As a result of the smaller sample size, only a few covariates were included in the statistical models to minimize overfitting, which could contribute to residual confounding. This limitation is further compounded by the short study duration (8 days), which restricts the ability to capture longer-term MVPA patterns and potential fluctuations due to external factors such as school schedules, weather changes, or social influences [75,78,95]. Future studies could benefit from extending the data collection period or conducting a longitudinal cohort study (>1 year) to improve statistical power and allow for stronger conclusions about causality and directionality. Additionally, randomized controlled trials could be used to evaluate the effectiveness of mEPAs in promoting physical activity, assessing whether real-time monitoring and feedback mechanisms lead to sustained behavioural changes. Leveraging performance-based incremental incentives for completing mEPA prompts could also enhance engagement and reduce attrition rates in future MVPA studies [108].” (lines 606 to 625).

We have also added to the Discussion: “The feasibility of implementing prospective digital assessments at scale may be influenced by disparities in smartphone access and internet connectivity, which could limit participation and introduce selection bias [104–106]. Future research should examine how differences in technology access impact participation and data quality. Additionally, exploring alternative delivery methods, such as hybrid digital and low-bandwidth approaches could help improve accessibility and ensure broader participation in digital PA monitoring [106]. In addition to access challenges, despite increasing digital literacy levels overall, participants with lower digital literacy may struggle with app navigation, leading to missing or inconsistent data [106,107]. Providing user-friendly interfaces and technical support could help mitigate these issues. Sustained participant engagement is another key consideration, as compliance with mEPAs may decline over time due to notification fatigue, competing priorities, and survey burden [20,21]. Research on optimizing adherence strategies, such as gamification, adaptive prompting, or incentives, could enhance long-term engagement in digital PA monitoring.” (lines 587 to 599).

6. Future Research Directions: Recommendation: Propose specific research questions or study designs that could address the limitations of the current study, such as randomized controlled trials to test the effectiveness of mEPAs in promoting physical activity.

Thank you for your feedback. We have now added to the Strengths and Limitations: “Future studies could benefit from extending the data collection period or conducting a longitudinal cohort study (>1 year) to improve statistical power and allow for stronger conclusions about causality and directionality. Additionally, randomized controlled trials could be used to evaluate the effectiveness of mEPAs in promoting physical activity, assessing whether real-time monitoring and feedback mechanisms lead to sustained behavioural changes.” (lines 618 to 623).

References

1. Inconsistent Formatting: For example, journal titles like "Progress in Cardiovascular Diseases" (Line 691) are not consistently italicized, and some references lack proper title case.

Thank you for your feedback. We have now proof-read the article to ensure the journals are consistently italicized and other references follow proper title case.

2. Missing Access Dates for Online Sources: Some references that include URLs (e.g., Lines 686 and 713) lack access dates.

Thank you for your comment. We have ensured that all references that include URLs

include access dates.

3. Redundant Citations: There are redundant citations of the same source, particularly for studies by Katapally TR (e.g., Lines 754, 795, and 829). These could be combined or referenced more efficiently to avoid repetition.

Thank you for your suggestion. We have reviewed the citations and streamlined them where possible. However, some references from the same author serve distinct purposes. For example, Line 754 describes the methodology for collecting prospective physical activity data, Line 795 discusses the longitudinal study, and Line 829 provides additional details on the Smart study not covered in the other sources. Each citation is included to ensure clarity and provide the necessary context for readers.

4. Citation Order: The references are not consistently ordered in the correct sequence based on their appearance in the text. For example, reference 51 (Line 830) appears before references 39 and 40 (Lines 795 and 799). Ensuring the correct order of citations is essential for coherence.

Thank you for your feedback. We have now ensured that the order of citations is in the correct sequence.

5. Incomplete References: A few citations lack complete information. For instance, Line 998 cites Van Domelen DR's study but does not provide a DOI or complete publication details. This could hinder readers from locating the source.

Thank you for your comment. We have ensured that all peer-reviewed articles have an associated DOI and relevant information for readers to easily locate the source.

Reviewer 2: Writing changes

Thank you for your suggestions. We have modified each of the sentences to align with the feedback provided.

- Lines 52–53: Physical inactivity is the fourth leading risk factor of mortality globally, which could be corrected as follows: Physical inactivity is the fourth leading risk factor for mortality worldwide.
- Lines 55–56: As digital citizen science approaches utilizing citizen-owned smartphones to ethically obtain MVPA big data can transform physical activity (PA) surveillance..., can be corrected to Digital citizen science approaches using citizen-owned smartphones to collect MVPA data ethically have the potential to transform physical activity (PA) surveillance.
- Lines 57–58: ...using smartphone-deployed retrospective validated surveys (adapted from the International Physical Activity Questionnaire, the Simple Physical Activity Questionnaire, and the Global Physical Activity Questionnaire)... cam be corrected to...using smartphone-deployed, validated retrospective surveys adapted from the International Physical Activity Questionnaire (IPAQ), Simple Physical Activity Questionnaire (SPAQ), and Global Physical Activity Questionnaire (GPAQ)...
- Line 62: A total of 68 participants (aged 13 to 21) provided complete data... could be correct to, Sixty-eight participants (aged 13–21) provided complete data... (I prefer spelling out numbers at the beginning of a sentence.)
- Line 103: Evidence is clear that physical activity (PA) is crucial in the prevention and management of non-communicable diseases (NCD) among youth. Can be changed to The evidence clearly indicates that physical activity (PA) is crucial for the prevention and management of non-communicable diseases (NCDs) among youth.
- Line 106: ...evidence indicating the presence of a dose-response relationship between PA and health – the more PA that youth engage in, the greater their health benefits. This can be corrected to ...evidence suggests a dose-response relationship between PA and health; the more youth engage in PA, the greater the health benefits.
- Line 110: Additionally, engagement in consistent PA has been found to improve sleep quality, reduce symptoms of depression and anxiety, and minimize substance use. Try this version, Engaging in regular PA has been shown to improve sleep quality, reduce symptoms of depression and anxiety, and decrease substance use.
- Line 121: Measuring MVPA frequency enables direct comparisons with established guidelines, making it easier to assess adherence to recommended levels of PA. can be corrected to: Measuring the frequency of MVPA allows for direct comparisons with established guidelines, facilitating the assessment of adherence to recommended PA levels.
- Line 125: Recall errors introduce inaccuracies in reported MVPA data, while the influence of social desirability, particularly apparent in subjective recall measures, can further distort the reliability of MVPA data. To. : Recall errors introduce inaccuracies in reported MVPA data, and the influence of social desirability, especially in subjective recall measures, can further compromise data reliability.
- Line 133: Repeated prospective measures can address these deficiencies of existing methodologies, particularly when deployed via citizen-owned digital devices to minimize recall bias and enhance collection of environmental factors that influence MVPA. Can be Repeated prospective measures can address the limitations of existing methodologies, especially when implemented through citizen-owned digital devices, reducing recall bias and improving the capture of environmental factors affecting MVPA.
- Line 171: To address these concerns, we modified the momentary assessments to encompass a longer reporting interval (i.e., end of day), following Shiffman et al. can be corrected: To address these issues, we adapted the momentary assessments to include a longer reporting interval (i.e., end of day), following the approach of Shiffman et al.
- Line 191: ...in Regina, an urban centre located in the Canadian prairie province of Saskatchewan. I recommend to this change ...in Regina, an urban center in the Canadian prairie province of Saskatchewan.
- Line 199: Ethics approval for the Smart Platform was granted by the Research Ethics Board of the University of Regina and Saskatchewan. I recommend to this change Ethical approval for the Smart Platform was granted by the Research Ethics Boards of the University of Regina and Saskatchewan .
- Line 212: Through the app, participants reported their MVPA data, behavioural, contextual, demographic and social factors influencing their PA behaviour. I recommend to this change Through the app, participants reported their MVPA data, as well as behavioral, contextual, demographic, and social factors influencing their PA behavior.
- Line 218: Additionally, they were asked to provide responses to mEPAs from day 1 to day 8, including weekdays and weekends through an in-app time-triggered nudge. I recommend to this change They were also prompted to provide responses to mEPAs from day 1 to day 8, including both weekdays and weekends, via an in-app time-triggered nudge.
- Line 230: By conducting a sample size calculation at a 90% confidence level with a 5% margin of error, it was determined that a sample size of 273 participants was required for the study. I recommend to this change A sample size calculation at a 90% confidence level with a 5% margin of error determined that 273 participants were required.
- Line 238: During these recruitment sessions, the research team described the study, demonstrated how to use the custom-built app, which included demonstrations of completing mEPAs among other tasks. I recommend to this change During these recruitment sessions, the research team explained the study, demonstrated how to use the custom-built app, including how to complete mEPAs and other tasks.
- Line 244: Implied informed consent was obtained from parents and caregivers of participants who were between 13 and 16 years old prior to the in-person scheduled recruitment sessions. I recommend to this change Implied informed consent was obtained from parents and caregivers of participants aged 13 to 16 before the in-person recruitment sessions.
- Line 251: The retrospective MVPA data were collected through a survey adapted from three validated self-reported measures: the international physical activity questionnaire, the simple physical activity questionnaire, and the global physical activity questionnaire. I recommend to this change Retrospective MVPA data were collected using a survey adapted from three validated self-reported measures: the International Physical Activity Questionnaire (IPAQ), Simple Physical Activity Questionnaire (SPAQ), and Global Physical Activity Questionnaire (GPAQ).
- Line 309: Peer support for MVPA was captured with the question: “How many of your closest friends are physically active?” can be changed to: Peer support for MVPA was assessed with the question: “How many of your closest friends are physically active?”
- Line 363: On how many days in the last 7 days did you do exercises to strengthen or tone your muscles? To: On how many days in the past 7 days did you engage in exercises to strengthen or tone your muscles?
- Line 385: Chi-square tests were used to test the significance difference in a proportion of independent variables included in this study. To : Chi-square tests were used to assess the significance of differences in the proportions of independent variables included in this study.
- Line 391: Covariates including peer and family support, strength training, distance and friends' drug-free were included in this study based in previous related literatures. To: Covariates, including peer and family support, strength training, distance, and friends' commitment to being drug-free, were included based on previous related literature.
- Line 396: This study involved the participation of 808 youth (13-21 years); however, when the cross-sectional survey was deployed at baseline, 436 participants provided complete data using the retrospective measure for capturing MVPA. Correction: This study included 808 youth (aged 13-21 years); however, only 436 participants provided complete data using the retrospective MVPA measure at baseline.
- Line 401: Youth were predominantly males (40.30%) of youth, with 56.72% being females, and 2.98% of them reported being transgender, other, or preferring not to reveal. Correction: The participants were predominantly female (56.72%), with 40.30% identifying as male, and 2.98% identifying as transgender, other, or preferring not to disclose.
- Line 416: Youth reported engaging in MVPA for an average of 5.06 times per week retrospectively and 2.10 times per week prospectively. Correction: Youth reported an average MVPA frequency of 5.06 times per week using the retrospective measure and 2.10 times per week using the prospective (mEPA) measure.
- Line 433: With respect to family support for MVPA, youth with a family member who encouraged them to participate in PA 5 to 6 times per week reported a higher frequency of MVPA than youth whose family members encouraged MVPA 4 times or less per week. Correction: Regarding family support for MVPA, youth whose family members encouraged PA 5 to 6 times per week reported a higher MVPA frequency compared to those whose family members encouraged PA 4 times or less per week.
- Line 448: Youth whose distance from school to their home was 5 km or more reported a higher frequency of MVPA in comparison to youth whose school is less than 5 km from their homes. Correction: Youth who lived 5 km or more from their school reported a higher frequency of MVPA compared to those who lived less than 5 km from their school.
- Line 453: The Poisson regression analyses showing the association between retrospective MVPA frequency (model 1) and prospective MVPA frequency (model 2) with sociodemographic and contextual factors are presented in Table 3. Correction: Table 3 presents the Poisson regression analyses showing the associations between sociodemographic and contextual factors with retrospective MVPA frequency (model 1) and prospective MVPA frequency (model 2).
- Line 488: This study was carried out to ascertain if there is a significant difference between the frequency of MVPA assessed through a digitally deployed retrospective survey... Correction: This study aimed to determine whether there is a significant difference in the frequency of MVPA assessed using a digitally deployed retrospective survey...
- Line 496: The main finding of the study was that there was a significant difference between the frequency of MVPA reported retrospectively and the frequency of MVPA reported via mEPAs. Correction: The main finding of the study was a significant difference in the frequency of MVPA reported retrospectively compared to that reported via mEPAs.
- Line 508: Social desirability bias is the propensity to self-report behaviour in a way that is viewed as desirable by society. Correction: Social desirability bias refers to the tendency to self-report behavior in a manner perceived as socially acceptable.
- Line 516: Near real-time data collection minimizes the potential distortion in data reporting that may arise from retrospective recall bias. Correction: Near-real-time data collection reduces the potential distortions in data reporting caused by retrospective recall bias.
